# Acute myocardial infarction in the Covid-19 era: Incidence, clinical characteristics and in-hospital outcomes—A multicenter registry

**DOI:** 10.1371/journal.pone.0253524

**Published:** 2021-06-18

**Authors:** Alexander Fardman, Doron Zahger, Katia Orvin, Daniel Oren, Natalia Kofman, Jameel Mohsen, Or Tsafrir, Elad Asher, Ronen Rubinshtein, Jafari Jamal, Roi Efraim, Majdi Halabi, Yacov Shacham, Lior Henri Fortis, Tal Cohen, Robert Klempfner, Amit Segev, Roy Beigel, Shlomi Matetzky

**Affiliations:** 1 Lev Leviev Heart and Vascular Center, Sheba Medical Center, Tel Hashomer, Israel; 2 Sackler School of Medicine, Tel Aviv University, Tel Aviv, Israel; 3 Department of Cardiology, Soroka University Medical Center, Beer Sheva, Israel; 4 Faculty of Health Sciences, Ben Gurion University of the Negev, Beer Sheva, Israel; 5 Department of Cardiology, Rabin Medical Center, Petach-Tikva, Israel; 6 Department of Cardiology, Shamir Medical Center, Tzrifin, Israel; 7 Department of Cardiology, Hillel Yaffe Medical Center, Hadera, Israel; 8 Ruth and Bruce Rappaport Faculty of Medicine, Technion, Israel Institute of Technology, Haifa, Israel; 9 Division of Cardiology, Galilee Medical Center, Nahariya, Israel; 10 Faculty of Medicine in the Galilee, Bar Ilan University, Safed, Israel; 11 The Jesselson Integrated Heart Center, Shaare Zedek Medical Center, Jerusalem, Israel; 12 Faculty of Medicine, Hebrew University, Jerusalem, Israel; 13 Department of Cardiology, Wolfson Medical Center, Holon, Israel; 14 Cardiology Department, Barzilai University Medical Center, Ashkelon, Israel; 15 Department of Cardiology, Rambam Healthcare Campus, Haifa, Israel; 16 Department of Cardiology, Ziv Medical Center, Safed, Israel; 17 Department of Cardiology, Tel-Aviv Sourasky Medical Center, Tel-Aviv, Israel; 18 Department of Cardiology, Samson Assuta Ashdod University Hospital, Ashdod, Israel; Erasmus Medical Centre: Erasmus MC, NETHERLANDS

## Abstract

**Background:**

We aimed to describe the characteristics and in-hospital outcomes of ST-segment elevation myocardial infarction (STEMI) patients during the Covid-19 era.

**Methods:**

We conducted a prospective, multicenter study involving 13 intensive cardiac care units, to evaluate consecutive STEMI patients admitted throughout an 8-week period during the Covid-19 outbreak. These patients were compared with consecutive STEMI patients admitted during the corresponding period in 2018 who had been prospectively documented in the Israeli bi-annual National Acute Coronary Syndrome Survey. The primary end-point was defined as a composite of malignant arrhythmia, congestive heart failure, and/or in-hospital mortality. Secondary outcomes included individual components of primary outcome, cardiogenic shock, mechanical complications, electrical complications, re-infarction, stroke, and pericarditis.

**Results:**

The study cohort comprised 1466 consecutive acute MI patients, of whom 774 (53%) were hospitalized during the Covid-19 outbreak. Overall, 841 patients were diagnosed with STEMI: 424 (50.4%) during the Covid-19 era and 417 (49.6%) during the parallel period in 2018. Although STEMI patients admitted during the Covid-19 period had fewer co-morbidities, they presented with a higher Killip class (p value = .03). The median time from symptom onset to reperfusion was extended from 180 minutes (IQR 122–292) in 2018 to 290 minutes (IQR 161–1080, p < .001) in 2020. Hospitalization during the Covid-19 era was independently associated with an increased risk of the combined endpoint in the multivariable regression model (OR 1.65, 95% CI 1.03–2.68, p value = .04). Furthermore, the rate of mechanical complications was four times higher during the Covid-19 era (95% CI 1.42–14.8, p-value = .02). However, in-hospital mortality remained unchanged (OR 1.73, 95% CI 0.81–3.78, p-value = .16).

**Conclusions:**

STEMI patients admitted during the first wave of Covid-19 outbreak, experienced longer total ischemic time, which was translated into a more severe disease status upon hospital admission, and a higher rate of in-hospital adverse events, compared with parallel period.

## Introduction

Since the beginning of the novel, coronavirus (SARS-CoV2) pandemic in December 2019 a number of studies from Europe [1–4], the United States [5] and Hong-Kong [6] have reported significant reductions in acute myocardial infarction (AMI) hospitalizations and catheterization laboratory activation rates. However, comprehensive data regarding the impact of the Covid-19 outbreak on AMI patient characteristics, clinical presentation, and in-hospital outcomes are scarce [3, 7]. Furthermore, the explanation for the observed reduction in AMI admission rates remains speculative. Several theories have been postulated to explain this observation, including fear of contracting the virus in the hospital, reduced air pollution, limited physical activity due to social restrictions, and increased utilization of telemedicine to prevent the deterioration of chronic conditions and the need for hospitalizations [1, 2, 4–6, 8]. Contrary to this trend, previous studies have reported an increased incidence of AMI immediately following stressful situations such as earthquakes or terrorist attacks [9–11].

The aim of the current study was to describe trends regarding characteristics, clinical presentation, and in-hospital outcomes of ST-segment elevation myocardial infarction (STEMI) patients during the surge of the Covid-19 pandemic in a nationwide survey.

## Materials and methods

We conducted a prospective, multicenter, observational, nationwide study involving 13 medical centers across Israel aimed at evaluating consecutive patients with STEMI admitted to intensive cardiac care units (ICCUs) over an 8-week period during the initial Covid-19 outbreak starting from March 9, 2020 through April 30, 2020. Social distancing restrictions were announced in Israel on March 11, 2020. All participating centers have percutaneous coronary intervention facilities operating 24/7. Data were anonymously documented in each ICCU by the local coordinator and prospectively submitted into an electronic case report form. The Chaim Sheba Medical Center served as the coordinating center for this study. Data were checked for accuracy and out-of-range values by the coordinating center. The institutional review board of each participating center approved the study and waived the need for individual informed consent based on strict safeguarding of participant anonymity by de-identifying patients during database entry. The list of the institutional reviewer boards and approval numbers included in the S1 Appendix.

### Data collection

Patient data from the control period was obtained from the Acute Coronary Syndrome Israeli Survey (ACSIS). ACSIS is a biannual survey which has been conducted in all ICCUs in Israel during March-April by the Israeli Working Group on Acute Cardiac Care of the Israel Heart Society since 2000. During these two-monthly periods, detailed data regarding all consecutive AMI patients are prospectively collected in all ICCUs and cardiology wards in all public hospitals in Israel. For the current study, we used data from the corresponding period in 2018 (March 9 through April 30) from 13 medical centers that participated in the 2020 survey (S2 Appendix). The incidence of newly confirmed coronavirus cases in Israel was based on the Ministry of Health records.

We recorded the number of patients admitted to each ICCU with STEMI and non-STEMI (NSTEMI) during the above-named period. The diagnosis of AMI was defined as clinical symptoms compatible with myocardial ischemia and troponin elevation. STEMI was diagnosed according to the contemporary guidelines [12], otherwise NSTEMI was diagnosed. For each patient admitted with STEMI, we collected demographic data, clinical characteristics, main angiographic, and echocardiographic parameters. Time from symptom onset to hospital admission and time from hospital admission to reperfusion (defined at the time of guidewire passage), were documented for each STEMI patient. Patients’ clinical status on hospital admission was assessed by the Killip classification.

The following in-hospital complications were recorded: sustained ventricular arrhythmia, congestive heart failure defined as pulmonary congestion requiring administration of intravenous diuretics, cardiogenic shock, cardiac arrest, mechanical complications (free-wall rupture, ventricular septal defect or moderate/severe mitral regurgitation), second degree or higher atrio-ventricular block, atrial fibrillation, re-infarction, stroke, mechanical ventilation, major bleeding, pericarditis and in-hospital mortality. The primary outcome of this study was a composite of sustained ventricular arrhythmia, pulmonary congestion, and/or in-hospital mortality.

### Statistical analysis

Categorical variables were compared by the chi-square test or Fisher exact test. The distribution pattern was tested with Shapiro-Wilk test. Normally distributed continuous variables were compared by the Student’s t-test. Non-normally distributed variables were compared using the Mann-Whitney-Wilcoxon test. Mean admission rates for AMI-related hospitalizations were calculated by dividing the number of cumulative events by the number of days for each time period. Incidence rate ratios (IRR) comparing the Covid-19 period (2020) to the control period (2018) were calculated using unadjusted Poisson regression to model the number of AMI-related hospitalizations per day in the entire cohort and in the STEMI sub-group. A multivariable logistic regression model adjusted to older age (>65 years), diabetes mellitus, hypertension, dyslipidemia, smoking status, prior coronary artery disease, and chronic renal failure was used to evaluate an association between the study period and combined outcome as well as its individual components. In addition, we divided our cohort into tertiles according to the total ischemic time, and examined the association between the study period and the upper tertile of total ischemic time, using a multivariable logistic regression model adjusted to the same parameters. A two-sided p-value of < .05 was considered statistically significant. All analyses were performed with R software version 3.4.4 (R Foundation for Statistical Computing).

## Results

The study cohort comprised 1466 patients, of whom 774 (52.8%) were hospitalized between March 9 and April 30, 2020, and 692 (47.2%) who were admitted during the corresponding period in 2018, representing a 12% increase in overall admissions for AMI during the Covid-19 era. There were 841 STEMI patients (58%), including 424 (50.4%) hospitalized during the Covid-19 era and 417 (49.6%) in 2018, representing a 1.7% increase in STEMI admissions during the Covid-19 era. Patient distribution according to the medical centers is presented in S1 Table.

### Acute myocardial infarction hospitalization rates and Covid-19 incidence

**Fig 1:**
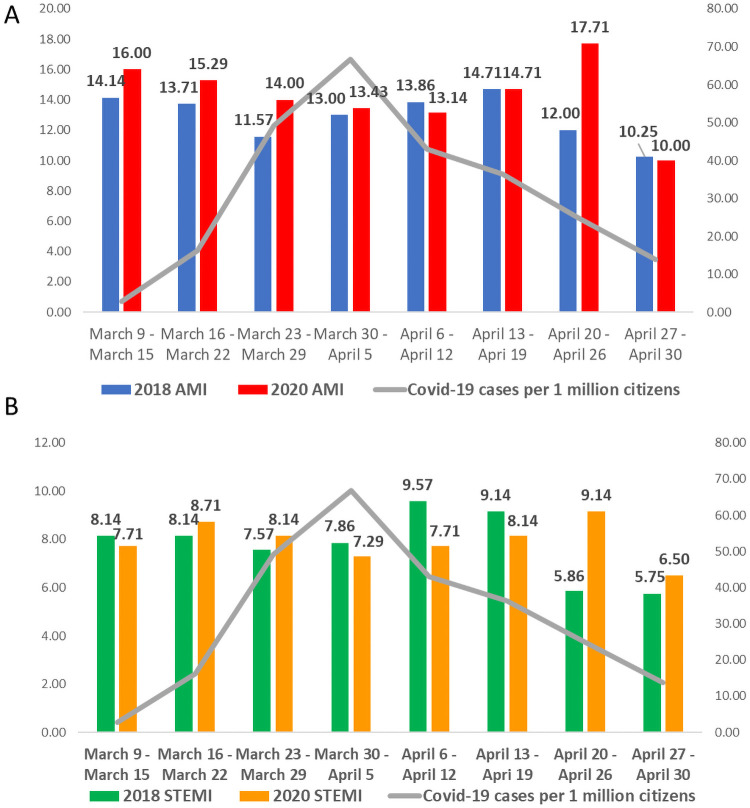
Daily incidence of hospitalized acute MI cases (A) and ST-segment elevation MI cases (B) before and during the Covid-19 pandemic and incidence of newly confirmed Covid-19 cases. Daily incidence of acute MI cases during 2018 (Fig 1A, blue bars) and during 2020 (Fig 1A, red bars); daily incidence of ST-segment elevation MI cases during 2018 (Fig 1B, green bars) and during 2020 (Fig 1B, yellow bars); daily incidence of newly confirmed Covid-19 cases per 1 million Israeli citizens (Fig 1A, 1B, grey line). MI = Myocardial infarction.

The mean overall daily AMI admission rate was 14.9 during the study period and 13.3 during the control period (IRR 1.1, 95% Confidence Interval [CI] 0.2–5.8, p-value = .91). Results remained similar when only STEMI admissions were analyzed: 8.15 during the study period and 8.02 during the control period (IRR 1.0, 95% Confidence Interval [CI] 0.2–6.0, p-value = .99). During the above-mentioned period in 2020, a daily ratio of newly confirmed coronavirus-infected cases ranged from 2.8 per 1 million inhabitants during the first week (March 9–March 15) to 13.8 per 1 million during the last week (April 27–April 30) (Fig 1). Results of SARS-CoV-2 testing were available for 194 (25.3%) AMI patients of which only one test was positive.

### Baseline characteristics

Baseline characteristics of STEMI patients are presented in Table 1. Approximately 80% were male and the median age was 62 years (IQR 54–71). There were no significant differences in age and gender between the two study periods. Notably, patients admitted during the Covid-19 era were significantly less likely to have dyslipidemia (58% vs 68%, p value = .003), prior MI (18% vs 27%, p value = .004) and prior stroke/transient ischemic attack (5.9% vs 10.6%, p value = .019), compared to those admitted during the control period.

**Table 1:** Baseline characteristics of STEMI patients before and during the Covid-19 pandemics.

Baseline characteristic	Total N = 841	Covid-19 era, N = 424	Control period, N = 417	P value
Age, years median (IQR)	62 (54,71)	62 (55,71)	61 (54,70)	.497
Female gender, N (%)	151 (18)	81 (19)	70 (17)	.432
Diabetes Mellitus, N (%)	287 (34)	132 (31)	155 (37)	.072
Hypertension, N (%)	460 (55)	220 (52)	240 (58)	.097
Dyslipidemia, N (%)	529 (63)	246 (58)	283 (68)	.003
Current smoker, N (%)	422 (50)	206 (49)	216 (52)	.388
Prior CKD, N (%)	60 (7.1)	23 (5.4)	37 (8.9)	.071
Prior MI, N (%)	188 (22)	77 (18)	111 (27)	.004
Prior CABG, N (%)	28 (3.3)	11 (2.6)	17 (4.1)	.314
Prior PAD, N (%)	36 (4.3)	12 (2.8)	24 (5.8)	.052
Prior CHF, N (%)	45 (5.4)	18 (4.2)	27 (6.5)	.199
Prior stroke/TIA, N (%)	69 (8.2)	25 (5.9)	44 (10.6)	.019

STEMI = ST-segment elevation myocardial infarction; IQR = Interquartile range; CKD = Chronic kidney disease; MI = Myocardial infarction; CABG = Coronary artery bypass grafting; PAD = peripheral artery disease; CHF = Congestive heart failure; TIA = transient ischemic attack.

### Clinical presentation

**Fig 2:**
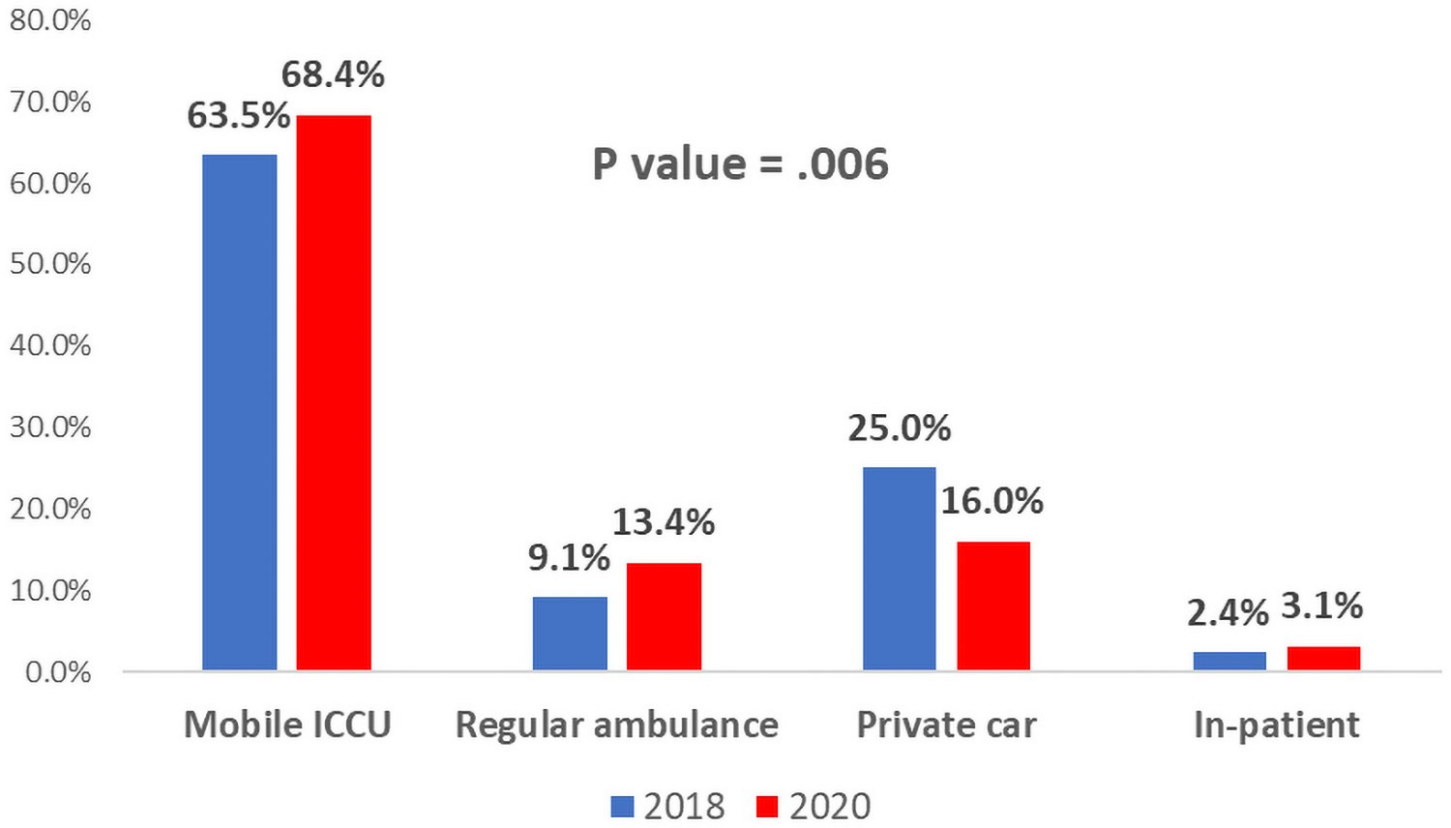
Distribution of STEMI patients according to the mode of transportation before and during the Covid-19 pandemic. Blue bars represent patients admitted with STEMI during the control period in 2018 and red bars represent patients admitted with STEMI during the first wave of Covid-19 (2020). ICCU = Intensive cardiac care unit; STEMI = ST-segment elevation myocardial infarction.

During the Covid-19 era, more patients arrived at the hospital via an emergency medical system (EMS) compared with the control period (82% vs. 72.6%, p value = .006) (Fig 2).

**Fig 3:**
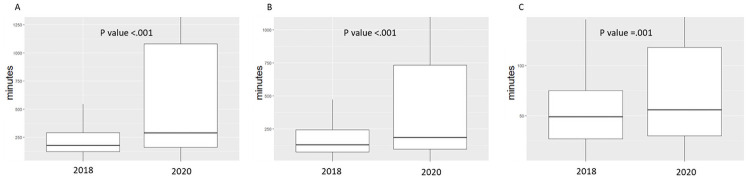
Distribution of total ischemic time and its individual components in STEMI patients before and during the Covid-19 pandemic. The graph represents median and interquartile range of time components from symptom onset to reperfusion therapy during the first wave of Covid-19 (2020) vs the control period (2018). A. Total ischemic time–time from symptom onset to reperfusion. B. Time from symptom onset to hospital admission. C. Time from hospital admission to reperfusion.

We observed significant differences between the time intervals of STEMI patients in the Covid-19 era group, and the control period (Table 2 and Fig 3). Time from symptom onset to hospital admission (patient delay) was extended from 130 minutes in 2018 (IQR 75–243) to 186 minutes (IQR 97–732, p-value < .001) in 2020. Similarly, time from hospital admission to reperfusion (system delay) was extended from 49 minutes (IQR 27–75) in 2018 to 56 minutes (IQR 30–118, p-value = .001) in 2020. Overall time from symptom onset to reperfusion (total ischemic time) was lengthened from 180 minutes (IQR 122–292) in 2018 to 290 minutes (IQR 161–1080, p value < .001) in 2020.

**Table 2:** Clinical presentation and angiographic findings in STEMI patients before and during the Covid-19 pandemic.

Characteristic	Total N = 841	Covid-19 era N = 424	Control period N = 417	P value
Time from symptom onset to hospital admission (minutes), median (IQR)	163 (89,464)	186 (97,732)	130 (75,243)	< .001
Time from hospital admission to reperfusion (minutes), median (IQR)	52 (29,90)	56 (30,118)	49 (27,75)	.001
Time from symptom onset to reperfusion (minutes), median (IQR)	230 (140,693)	290 (161,1080)	180 (122, 292)	< .001
Mode of transportation:				.006
Mobile ICCU, N (%)	555 (66)	290 (68.4%)	265 (63.5%)	
Regular ambulance, N (%)	95 (11.3)	57 (13.4%)	38 (9.1%)	
Private car, N (%)	172 (21)	68 (16%)	104 (25%)	
In-patient, N (%)	19 (2.3)	9 (3.1%)	10 (2.4%)	
Killip class at admission:				.030
1, N (%)	646 (85.3)	297 (81%)	349 (89%)	
2, N (%)	43 (5.7)	26 (7.1%)	17 (4.3%)	
3, N (%)	30 (4)	18 (4.9%)	12 (3.1%)	
4, N (%)	38 (5)	24 (6.6%)	14 (3.6%)	
Admission heart rate, bpm, median (IQR)	78 (69,90)	79 (68,90)	78 (67,90)	.525
Admission systolic BP, mmHg, mean ± SD	137 ± 28	135 ± 28	139 ± 29	.065
Admission diastolic BP, mmHg, median (IQR)	80 (70,90)	80 (70,90)	80 (71,92)	.341
Primary reperfusion, N (%)	698 (87)	368 (87)	330 (87)	.955
Total angiography during hospitalization, N (%)	822 (98)	420 (99)	402 (96)	.018
Number of diseased vessels:				< .001
1, N (%)	314 (39)	183 (44%)	131 (34%)	
2, N (%)	246 (31)	127 (30%)	119 (31%)	
3, N (%)	190 (24)	103 (24%)	87 (23%)	
MINOCA, N (%)	51 (6.4)	6 (1.4%)	45 (12%)	
Infarct Related Artery:				.202
Left main, N (%)	1 (0.1)	0 (0.0)	1 (0.3%)	
LAD, N (%)	340 (47)	196 (48.6%)	144 (45.3%)	
LCx, N (%)	104 (14.4)	65 (16.1%)	39 (12.3%)	
Ramus, N (%)	6 (0.8)	4 (1.0%)	2 (0.6%)	
RCA, N (%)	265 (37)	137 (34%)	128 (40.3%)	
Vein graft, N (%)	4 (0.6)	1 (0.2%)	3 (0.9%)	
EF, median (IQR)	45 (39,50)	45 (40,50)	45 (38,51)	.937
Length of hospitalization (days), median (IQR)	4 (3,5)	4 (3,5)	3 (3,5)	.292

STEMI = ST-segment elevation myocardial infarction; IQR = interquartile range; ICCU = intensive cardiac care unit; BP = Blood pressure; SD = Standard deviation; MINOCA = myocardial infarction with non-obstructive coronary arteries; LAD = left anterior descending artery; LCx = Left circumflex artery; RCA = right coronary artery; EF = ejection fraction.

The differences in the ischemic time remained consistent when the cohort was divided according to the month of admission (S2 Table), gender (S3 Table) and age (S4 Table). Patients who arrived at the hospital independently experienced a longer overall ischemic time compared to those transferred by an EMS (349 minutes [IQR 187–904] vs 213 minutes [IQR 135–638], p value = .001); this was similarly observed for patient delay and system delay (S5 Table). In a multivariable logistic regression model adjusted for relevant clinical variables, admission to the hospital with STEMI during the Covid-19 era was independently associated with later arrival to coronary angiography, (Odds ratio [OR] 2.98, 95% CI 1.78–5.14, p < .001) (S6 Table).

**Fig 4:**
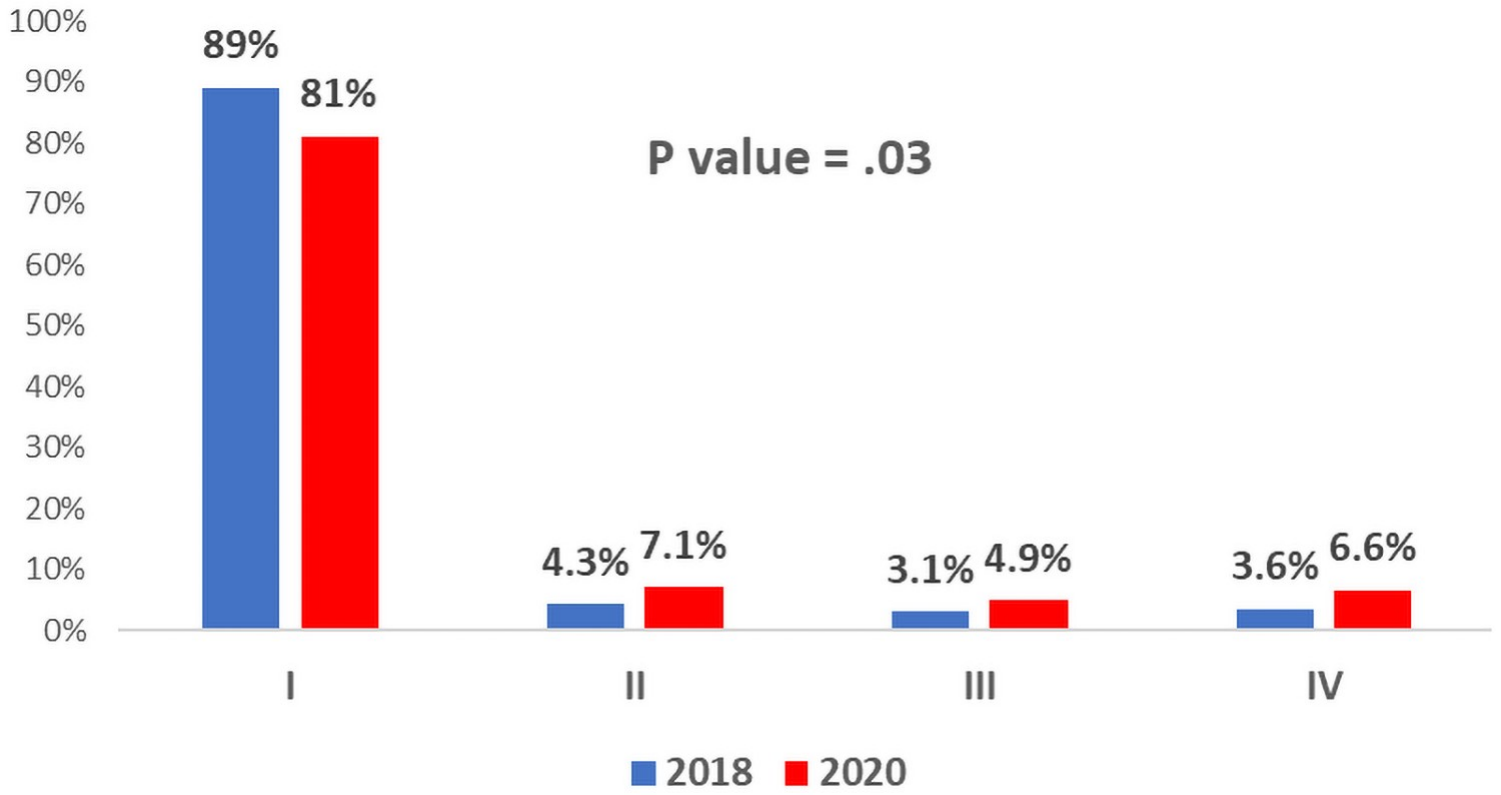
Distribution of STEMI patients according to Killip class at hospital admission before and during the Covid-19 pandemic. Red bars represent patients admitted with STEMI during the first wave of Covid-19 in 2020 and blue bars represent patients admitted with STEMI during the corresponding control period in 2018.

The Killip classification upon admission was available for 757 (90.01%) patients. Patients hospitalized during the Covid-19 era had a higher Killip class on arrival compared with patients admitted during the control period (p value = .03) (Fig 4). Notably, patients admitted during the Covid-19 era were almost twice more likely to present with severe hemodynamic compromise (Killip class III\IV) compared with patients in 2018 (11.5% vs. 6.6%, p-value = .027), despite a better profile of baseline comorbidities in the Covid-19 era.

### Angiographic findings and coronary revascularization

Overall, 822 (98%) patients admitted with STEMI underwent coronary angiography during hospitalization. While the proportion of patients who underwent primary reperfusion was similar in both study groups (87% vs 87%, p-value = .955), more patients underwent coronary revascularization during their in-hospital course during the Covid-19 era (99% vs. 96%, p-value = .018). Interestingly, angiographic findings differed between the two study groups. The rate of myocardial infarction non-obstructive coronary arteries (MINOCA) was eight times higher in the control period (11.8%), compared with the Covid-19 era (1.4%), while single-vessel disease was the most common angiographic finding in the Covid-19 era (44% vs. 34%, p value < .001). Complete angiographic characteristics were available for 721 (85.7%) patients (Table 2).

### In-hospital outcomes

**Fig 5:**
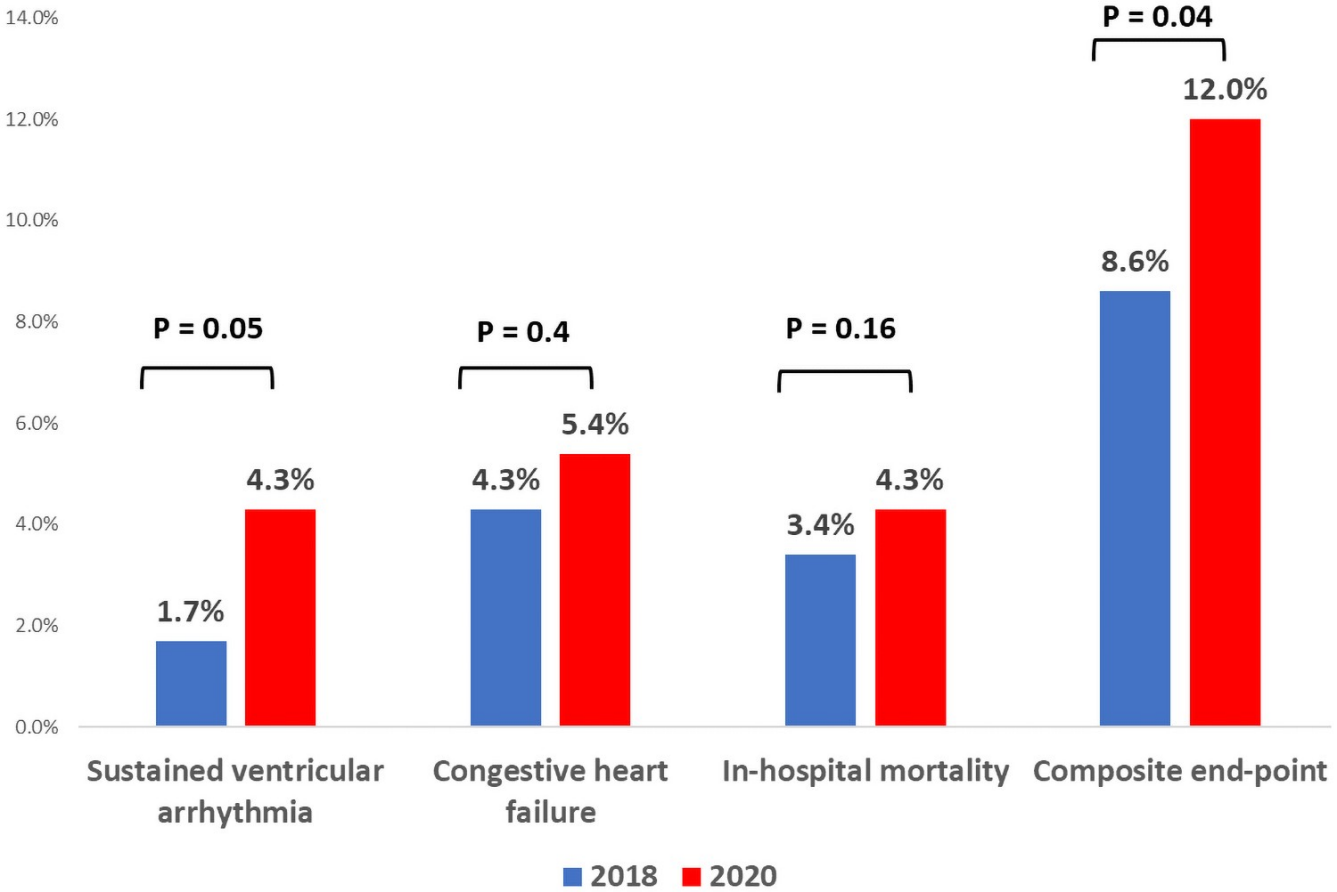
Distribution of primary end-point and its individual components according to the hospitalization period. Red bars represent patients admitted with STEMI during the Covid-19 era and blue bars represent patients admitted with STEMI during the corresponding control period in 2018. *Multivariable logistic regression model further adjusted for elderly patients (> 65 years), diabetes mellitus, hypertension, dyslipidemia, smoking status, prior coronary artery disease and chronic renal failure.

In-hospital outcomes were available for 99.4% of patients. As shown in Table 3, hospitalization during the Covid-19 period was independently associated with an increased risk for the prespecified combined in-hospital outcome (OR 1.65, 95% CI 1.03–2.68, p value = .04) (Fig 5). When the secondary outcomes were analyzed using the multivariable logistic regression model, admissions during the Covid-19 era were associated with a higher incidence of sustained ventricular arrhythmias (OR 2.51, 95% CI 1.05–6.67, p-value = .05) and mechanical complications (OR 4.09, 95% CI 1.42–14.8, p-value = .02). In addition, a numerically higher incidence of cardiac arrest (OR 2.11, 95% CI 1.00–4.65, p-value = .06) was recorded. The rates of re-infarction (OR 1.48, 95% CI 0.24–11.27, p-value = .67), stroke (OR 2.48, 95% CI 0.53–17.36, p-value = .29) and in-hospital mortality (OR 1.73, 95% CI 0.81–3.78, p-value = .16) were similar during the two study periods.

**Table 3:** Multivariable* logistic regression model for in-hospital outcomes of STEMI patients during the Covid-19 pandemic.

Outcome	Covid-19 era N = 424	Control period N = 417	Odds Ratio^†^	95% Confidence interval	P value
Combined in-hospital outcome^‡^	51 (12)	36 (8.6)	1.65	1.03–2.67	.04
Sustained ventricular arrhythmia	18 (4.2)	7 (1.7)	2.51	1.05–6.67	.05
Congestive heart failure	23 (5.4)	18 (4.3)	1.33	0.69–2.60	.40
Mechanical complications^#^	15 (3.5)	4 (1)	4.09	1.42–14.8	.02
High degree AVB	10 (2.4)	14 (3.4)	0.64	0.30–1.31	.23
Cardiogenic shock	27 (6.4)	23 (5.4)	1.31	0.72–2.4	.38
Cardiac arrest	20 (4.7)	13 (3.1)	2.11	1.00–4.65	.06
Atrial fibrillation	22 (5.2)	22 (5.3)	0.89	0.47–1.67	.71
Pericarditis	7 (1.6)	4 (1)	1.52	0.44–6.04	.53
Re-infarction	3 (0.7)	2 (0.5)	1.48	0.24–11.27	.67
Stroke	5 (1.2)	2 (0.5)	2.48	0.53–17.36	.29
Mechanical ventilation	30 (7.1)	29 (7.0)	1.28	0.73–2.24	.40
Major bleeding	3 (0.7)	8 (1.9)	0.45	0.09–1.68	.27
In-hospital mortality	18 (4.3)	14 (3.4)	1.73	0.81–3.78	.16

*The model is further adjusted for elderly patients (> 65 years), diabetes mellitus, hypertension, dyslipidemia, smoking status, prior coronary artery disease and chronic renal failure.

^†^Odds ratio to develop pre-defined outcome during the Covid-19 era using the 2018 period as a reference.

^‡^Combined in-hospital outcome was defined as a composite of sustained ventricular arrhythmia, pulmonary congestion, and/or in-hospital mortality.

^#^ Free-wall rupture, ventricular septal defect, moderate/severe mitral regurgitation.

AVB = atrio-ventricular block; STEMI = ST-segment elevation myocardial infarction.

We found no significant differences in the distribution of anti-thrombotic, lipid lowering and novel glucose lowering medications upon discharge from the index hospitalization (S7 Table).

## Discussion

In this multicenter prospective study, we describe a population of STEMI patients admitted to ICCUs during the first wave of Covid-19 in 2020, provide patients’ clinical characteristics, describe their in-hospital course, and compare them to the historical cohort from the corresponding period in 2018. We demonstrate in a prospective manner a significant worsening of the prognosis of STEMI patients admitted during the Covid-19 pandemic.

Contrary to recent studies which have suggested a 25–40% reduction in the rate of hospitalized acute coronary syndrome cases during the Covid-19 outbreak [1–6, 13, 14], we did not detect a significant difference in the rate of STEMI admissions compared with the corresponding period in 2018. The reported reduction in the incidence of hospitalizations for AMI during the Covid-19 outbreak was also contrary to prior studies which showed that psychosocial stress was associated with an increased risk of AMI [15]. Natural disasters and terrorist attacks have also been associated with an increase in cardiovascular events [10, 11]. Similarly, stressful circumstances, resulting from restrictions in freedom of movement, economic uncertainty and changes in social infrastructure occurring during global pandemics, may result in an increase in AMI incidence.

There are several possible explanations for the difference between prior studies [1–6, 13, 14], which showed a reduction in AMI hospitalizations during the Covid-19 outbreak, and our results. First, the decline in admission rates of AMI patients during the pandemic is not uniformly distributed around the globe. In the international survey conducted by the European Society of Cardiology in 141 countries, 17% of healthcare professionals suggested that there had been no change or even an increase in STEMI presentation rates during the Covid-19 pandemic [16]. Secondly, it has been previously described that financial concerns may alter the decision to seek medical care in AMI patients with inadequate health care insurance coverage [17]. Economic instability resulting from current pandemics might therefore cause uninsured patients to opt to remain at home from fear of the high-cost burden. In contrast, public mandatory health coverage in Israel eliminates lack of insurance concerns. Lastly, a probable impact of seasonal variations in AMI incidence was accounted for by comparing with a control group from the corresponding months in 2018, contrary to studies that compared with the earlier period from the same year [1, 13].

In the current study, the Covid-19 cohort was characterized by a lower burden of cardiovascular comorbidities and history of prior MI and cerebrovascular events, compared with historical controls. This finding supports those of a previous study which found a similar difference in the rate of prior MI in patients presenting with AMI during the Covid-19 pandemic [7]. Cardiovascular risk factors such as hypertension, diabetes and prior coronary artery disease are associated with the development of severe Covid-19 illness [18]. This association has been highlighted by the media. Consequently, patients with multiple comorbidities may defer hospital admission, providing a possible explanation for the lower prevalence of cardiovascular risk factors and prior cardiovascular events among STEMI patients hospitalized during the Covid-19 era.

A small report from a single center in Hong-Kong described a worrisome increase in total ischemic time as well as in its components in 7 AMI patients during the Covid-19 pandemic [6]. We substantially expanded this finding in a large multicenter registry. In the current study, both patient-related and system-related delays were significantly prolonged during the Covid-19 era leading to more than a 50% increase in total ischemic time in STEMI patients. These results were not influenced by gender, age group or the month of admission. Furthermore, the Covid-19 era was independently associated with a longer ischemic time in a multivariable model. The delay is even more noteworthy given the increased use of EMS as a transportation strategy during the Covid-19 era, as there is a strong association between EMS transportation and earlier delivery of reperfusion therapy [19]. Patient delay translated into a higher proportion of patients presenting with pulmonary edema and cardiogenic shock (Killip score > 2) upon hospital arrival. In contrast to our findings, Mesnier and colleagues did not detect any difference in time interval from symptom onset to hospital arrival in STEMI patients admitted after lockdown, compared with patients admitted before lockdown. However, time from hospital arrival to reperfusion, a major determinant of in-hospital outcomes in patients with STEMI, was not reported in this study [13]. Interestingly, a previous study reported a higher rate of in-hospital ACS and lower rate of angiographically detectable culprit lesions in 2020, compared with historical controls [14]. We could not detect any difference in the rate of in-hospital ACS, but did find a similar rate of primary reperfusion performed in both cohorts. These discrepancies could be related to a higher proportion of Covid-19 positive patients and inclusion patients with unstable angina in the previous study [14].

In the current study we a found similar rate of in-hospital mortality during the first wave of Covid-19, compared to a parallel time frame in 2018. This finding is in line with the previous multicenter study from Spain that reported similar 30-day mortality rate during 2020 as compared with 2019 [14]. On the contrary, in hospital complications and mortality rate in the sub-group of Covid-19 positive ACS patients were significantly higher, compared to Covid-19 negative patients admitted during 2020, despite the comparable ischemic time [20]. In the current study we detected only one Covid-19 positive patient out of 194 (25.3%) patients tested, and therefore we could not include this in our discussion.

Our findings reinforce and extend previous reports that described an increase in in-hospital adverse events among patients presenting with STEMI during the Covid-19 pandemic [2, 14, 20]. While previous studies detected a higher rate of complications mainly in Covid-19 positive ACS patients, in the current report the adverse in-hospital course is unrelated to underlying infection [2, 14, 20]. We report a higher incidence of major in-hospital outcomes, including a 65% increase in the rate of combined primary end-point, a 2-fold increase in sustained ventricular arrhythmia and a 4-fold increase in the mechanical complications rate. Additionally, we noticed a 2-fold numerical increase in the incidence of cardiac arrest during 2020. This tendency was observed despite the comparable rate of primary reperfusion procedures during both periods. Moreover, patients admitted during the Covid-19 era exhibited a lower burden of cardiovascular co-morbidities, compared with the historical cohort. Collectively, these findings probably reveal an association between delayed presentation and longer door-to-balloon time of STEMI patients and a complicated in-hospital course during the Covid-19 outbreak.

### Limitations

Several study limitations should be mentioned. First, this is an observational study reporting on associations, and thus no causation can be inferred. Second, our report reflects patients who arrived at the hospital alive and does not account for those who died at home. Third, results of Covid-19 testing were available for only a quarter of our cohort, and therefore we cannot exclude that an underlying coronavirus infection contributed to the incidence of AMI and worse clinical outcomes in the current study. Fourth, we could not assess the exact time point of EMS activation and, therefore, could not assess an EMS delay. Fifth, due to substantial diversity in troponin assays across medical centers we did not collect troponin levels of study participants. Finally, data collection is a challenging process during the surge of a global pandemic and therefore several parameters, such as Killip class upon hospital arrival and angiographic properties were not available for the entire cohort.

## Conclusions

The current study provides an in-depth prospective insight into the characteristics, management and clinical outcomes of consecutive patients presenting with STEMI during the first wave of Covid-19 pandemic. Although in-hospital mortality remained unchanged during this time period, prolonged ischemic time, higher Killip class upon hospital arrival and a higher rate of in-hospital adverse events underscore the collateral damage of the pandemics and the need for a comprehensive strategy that will address both patient and system-related determinants of care in order to face future outbreaks should they occur. Policy makers and social media should be recruited to deliver the message that hospitals remain the best place for acute care of AMI patients, and educate the general public regarding symptoms that must trigger prompt action in seeking medical help.

## Supporting information

S1 Appendix Ethics statement. (DOCX)

S2 Appendix List of participating hospitals. (DOCX)

S1 Table AMI admitted before and during the Covid-19 era divided by the participating centers. (DOCX)

S2 Table Total ischemic time and its components before and during the Covid-19 era divided according to the admission month. (DOCX)

S3 Table Total ischemic time and its components before and during the Covid-19 era divided according to the patients’ gender. (DOCX)

S4 Table Total ischemic time and its components before and during the Covid-19 era divided according to the patients’ age. (DOCX)

S5 Table Total ischemic time and its components before and during the Covid-19 era divided by the mode of transportation. (DOCX)

S6 Table Multivariable logistic regression model for higher total ischemic time. (DOCX)

S7 Table Medications upon hospital discharge in STEMI patients before and during the Covid-19 pandemics. (DOCX)

S8 Table STROBE checklist for observational studies. (DOCX)
