# Supplementary material for: Acute myocardial infarction in the Covid-19 era: Incidence, clinical characteristics and in-hospital outcomes—A multicenter registry
Source: PLoS One. 2021 Jun 18;16(6):e0253524. doi: 10.1371/journal.pone.0253524 (PMC8213163; doi:10.1371/journal.pone.0253524)
Supplement: S1 Appendix — (DOCX) [file pone.0253524.s001.docx]

**S1 Appendix**

**Ethics statement**

The institutional review board of each participating center approved the study:

Chaim Sheba Medical Center, Tel Hashomer, Israel - 7067-20-SMC

Soroka Medical Center, Beer-Sheva, Israel – 0172-20

Rabin Medical Center, Petach-Tikva, Israel – 764-20

Shamir Medical Center, Tzrifin, Israel – ASF-0099-20

Hillel Yaffe Medical Center, Hadera, Israel – 144-20

Galilee Medical Center, Nahariya, Israel – 0064-20-NHR

Shaare Zedek Medical Center, Jerusalem, Israel – 0221-20-SZMC

Wolfson Medical Center, Holon, Israel – 0088-20

Barzilai Medical Center, Ashkelon, Israel – 0040-20-BRZ

Rambam Medical Center, Haifa, Israel – RMB-0257-20

Ziv Medical Center, Safed, Israel – 0037-20-ZIV

Tel-Aviv Medical Center, Tel-Aviv, Israel – 7067-20

Samson Assuta Ashdod Medical Center, Ashdod, Israel – 0048-20-AA
