# Supplementary material for: Acute myocardial infarction in the Covid-19 era: Incidence, clinical characteristics and in-hospital outcomes—A multicenter registry
Source: PLoS One. 2021 Jun 18;16(6):e0253524. doi: 10.1371/journal.pone.0253524 (PMC8213163; doi:10.1371/journal.pone.0253524)
Supplement: S2 Appendix — (DOCX) [file pone.0253524.s002.docx]

**S2 Appendix**

**List of participating hospitals**

**Leading study center**

Chaim Sheba Medical Center, Tel Hashomer, Israel

**Participating study centers**

Soroka Medical Center, Beer-Sheva, Israel

Rabin Medical Center, Petach-Tikva, Israel

Shamir Medical Center, Tzrifin, Israel

Hillel Yaffe Medical Center, Hadera, Israel

Galilee Medical Center, Nahariya, Israel

Shaare Zedek Medical Center, Jerusalem, Israel

Wolfson Medical Center, Holon, Israel

Barzilai Medical Center, Ashkelon, Israel

Rambam Medical Center, Haifa, Israel

Ziv Medical Center, Safed, Israel

Tel-Aviv Medical Center, Tel-Aviv, Israel

Samson Assuta Ashdod Medical Center, Ashdod, Israel
