# Supplementary material for: Acute myocardial infarction in the Covid-19 era: Incidence, clinical characteristics and in-hospital outcomes—A multicenter registry
Source: PLoS One. 2021 Jun 18;16(6):e0253524. doi: 10.1371/journal.pone.0253524 (PMC8213163; doi:10.1371/journal.pone.0253524)
Supplement: S1 Table — (DOCX) [file pone.0253524.s003.docx]

**S1 Table. AMI admitted before and during the Covid-19 era divided by the participating centers**

| **Participating center** | **Covid-19 era** | | **Control period** | |
| --- | --- | --- | --- | --- |
|  | **AMI** | **STEMI** | **AMI** | **STEMI** |
| Sheba MC | 75 | 48 | 77 | 30 |
| Shamir MC | 73 | 43 | 67 | 48 |
| Barzilai MC | 49 | 21 | 26 | 15 |
| Galilee MC | 66 | 33 | 48 | 36 |
| Rambam MC | 37 | 33 | 18 | 15 |
| Hillel Yaffe MC | 71 | 32 | 73 | 19 |
| Tel-Aviv Souraski MC | 34 | 27 | 44 | 39 |
| Shaare Zedek MC | 64 | 37 | 41 | 33 |
| Samson Assuta MC | 23 | 9 | 32 | 16 |
| Soroka MC | 103 | 64 | 78 | 66 |
| Ziv MC | 37 | 13 | 37 | 17 |
| Wolfson MC | 59 | 33 | 55 | 39 |
| Rabin MC | 83 | 31 | 96 | 44 |
| Total | 774 | 424 | 692 | 417 |

AMI= acute myocardial infarction; MC= Medical center; STEMI =St-segment elevation myocardial infarction.
