# Supplementary material for: Acute myocardial infarction in the Covid-19 era: Incidence, clinical characteristics and in-hospital outcomes—A multicenter registry
Source: PLoS One. 2021 Jun 18;16(6):e0253524. doi: 10.1371/journal.pone.0253524 (PMC8213163; doi:10.1371/journal.pone.0253524)
Supplement: S2 Table — (DOCX) [file pone.0253524.s004.docx]

**S2 Table. Total ischemic time and its components before and during the Covid-19 era divided according to the admission month**

| Characteristic | March | | | April | | |
| --- | --- | --- | --- | --- | --- | --- |
|  | Covid-19 era, N=182 | Control period, N=180 | P value | Covid-19 era, N=242 | Control period, N=237 | P value |
| Time from symptom onset to hospital admission (minutes), median (IQR) | 180 (96,776) | 126 (90,239) | .003 | 209 (98,700) | 132 (71,259) | <.001 |
| Time from hospital admission to reperfusion (minutes), median (IQR) | 54 (30,120) | 44 (28,77) | .012 | 57 (30,117) | 52 (27,72) | .022 |
| Time from symptom onset to reperfusion (minutes), median (IQR) | 258 (160,1067) | 180 (126,287) | <.001 | 300 (163, 1083) | 179 (120, 300) | <.001 |

IQR= interquartile range.
