# Supplementary material for: Acute myocardial infarction in the Covid-19 era: Incidence, clinical characteristics and in-hospital outcomes—A multicenter registry
Source: PLoS One. 2021 Jun 18;16(6):e0253524. doi: 10.1371/journal.pone.0253524 (PMC8213163; doi:10.1371/journal.pone.0253524)
Supplement: S3 Table — (DOCX) [file pone.0253524.s005.docx]

**S3 Table. Total ischemic time and its components before and during the Covid-19 era divided according to the patients’ gender**

| Characteristic | Male | | | Female | | |
| --- | --- | --- | --- | --- | --- | --- |
|  | Covid-19 era, N=343 | Control period, N=347 | P value | Covid-19 era, N=81 | Control period, N=70 | P value |
| Time from symptom onset to hospital admission (minutes), median (IQR) | 180.00 (90.00, 606.50) | 126.00 (70.75, 228.25) | <.001 | 368.00 (133.50, 1446.50) | 160.00 (86.00, 355.00) | .003 |
| Time from hospital admission to reperfusion (minutes), median (IQR) | 55.00 (30.00, 116.75) | 47.00  (27.00, 69.75) | <.001 | 57.00 (31.00, 118.00) | 56.00 (32.50, 90.50) | .825 |
| Time from symptom onset to reperfusion (minutes), median (IQR) | 269.50 (154.25, 992.25) | 175.00 (120.00, 290.00) | <.001 | 467.00 (211.00, 1228.00) | 192.00 (157.00, 300.00) | <.001 |

IQR= interquartile range.
