# Supplementary material for: Acute myocardial infarction in the Covid-19 era: Incidence, clinical characteristics and in-hospital outcomes—A multicenter registry
Source: PLoS One. 2021 Jun 18;16(6):e0253524. doi: 10.1371/journal.pone.0253524 (PMC8213163; doi:10.1371/journal.pone.0253524)
Supplement: S4 Table — (DOCX) [file pone.0253524.s006.docx]

**S4 Table. Total ischemic time and its components before and during the Covid-19 era divided according to the patients’ age**

| Characteristic | Age ≤ 65 | | | Age > 65 | | |
| --- | --- | --- | --- | --- | --- | --- |
|  | Covid-19 era, N=254 | Control period, N=254 | P value | Covid-19 era, N=170 | Control period, N=163 | P value |
| Time from symptom onset to hospital admission (minutes), median (IQR) | 180.00 (90.00, 607) | 119.00 (70, 212) | <.001 | \| 210.00 (120.50, 957.50) \|  \| \| --- \| --- \| | 182.50 (91.00, 270.25) | .007 |
| Time from hospital admission to reperfusion (minutes), median (IQR) | 52.00 (32.00, 117) | 46.00  (27.00, 69) | .002 | 59.00 (28.00, 119.00) | 53.00 (29.50, 77.00) | .136 |
| Time from symptom onset to reperfusion (minutes), median (IQR) | 260 (142, 877) | 155.00 (117.00, 250) | <.001 | 374.50 (181.50, 1266.25) | \|  \| 216.50 (145.50, 327.50) \| \| --- \| --- \| | <.001 |

IQR= interquartile range.
