# Supplementary material for: Acute myocardial infarction in the Covid-19 era: Incidence, clinical characteristics and in-hospital outcomes—A multicenter registry
Source: PLoS One. 2021 Jun 18;16(6):e0253524. doi: 10.1371/journal.pone.0253524 (PMC8213163; doi:10.1371/journal.pone.0253524)
Supplement: S5 Table — (DOCX) [file pone.0253524.s007.docx]

**S5 Table. Total ischemic time and its components before and during the Covid-19 era divided by the mode of transportation**

| Characteristic | Independently | | | EMS | | |
| --- | --- | --- | --- | --- | --- | --- |
|  | Covid-19 era, N=68 | Control period, N=104 | P value | Covid-19 era, N=347 | Control period, N=303 | P value |
| Time from symptom onset to hospital admission (minutes), median (IQR) | 332.00 (141.00, 1511.00) | 128.00 (59.50, 273.00) | <.001 | 180.00 (90.00, 626.00) | 130.00 (84.00, 235.00) | .001 |
| Time from hospital admission to reperfusion (minutes), median (IQR) | 91.00 (48.00, 200.75) | 64.00 (46.50, 85.50) | .007 | 51.50 (28.00, 101.25) | 43.50 (22.25, 64.75) | .001 |
| Time from symptom onset to reperfusion (minutes), median (IQR) | \| 600.00 (260.00, 2616.00) \|  \| \| --- \| --- \| | 200.00 (130.00, 465.00) | <.001 | 252.50 (150.75, 917.75) | 170.50 (120.25, 260.25) | <.001 |

EMS = emergency medical services; IQR= interquartile range.
