# Supplementary material for: Acute myocardial infarction in the Covid-19 era: Incidence, clinical characteristics and in-hospital outcomes—A multicenter registry
Source: PLoS One. 2021 Jun 18;16(6):e0253524. doi: 10.1371/journal.pone.0253524 (PMC8213163; doi:10.1371/journal.pone.0253524)
Supplement: S6 Table — (DOCX) [file pone.0253524.s008.docx]

**S6 Table. Multivariable logistic regression model for longer time^*^ from symptoms onset to reperfusion in the study cohort.**

| Outcome | Odds Ratio | 95% Confidence interval | P value |
| --- | --- | --- | --- |
| 2020 (vs 2018 as a reference) | 2.98 | 1.78-5.14 | <.001 |
| Age above 65 | 1.18 | 0.69-2.0 | .54 |
| Diabetes Mellitus | 2.07 | 1.21-3.54 | .01 |
| Hypertension | 0.99 | 0.58-1.69 | .98 |
| Dyslipidemia | 1.15 | 0.68-1.98 | .61 |
| Smoking status | 1.98 | 0.82-4.79 | .16 |
| Prior CAD | 0.68 | 0.36-1.22 | .22 |

*Defined as upper tertile of time from symptoms onset to reperfusion

CAD = coronary artery disease
