# Supplementary material for: Acute myocardial infarction in the Covid-19 era: Incidence, clinical characteristics and in-hospital outcomes—A multicenter registry
Source: PLoS One. 2021 Jun 18;16(6):e0253524. doi: 10.1371/journal.pone.0253524 (PMC8213163; doi:10.1371/journal.pone.0253524)
Supplement: S7 Table — (DOCX) [file pone.0253524.s009.docx]

**S7 Table. Medications upon hospital discharge in STEMI patients before and during the Covid-19 pandemics**

| Baseline characteristic | Total, N=841 | Covid-19 era, N=424 | Control period, N=417 | P value |
| --- | --- | --- | --- | --- |
| Acetylsalicylic acid, N (%) | 777 (92) | 396 (93) | 381 (91) | .034 |
| P2Y12 inhibitor, N (%)  Clopidogrel, N (%)  Prasugrel, N (%)  Ticagrelor, N (%) | 769 (91)  152 (18)  318 (38)  299 (36) | 387 (91)  71 (17)  157 (37)  159 (38) | 382 (92)  81 (19)  161 (39)  140 (34) | 1  .36  .69  .25 |
| Statin, N (%) | 790 (94) | 400 (94) | 390 (94) | 1 |
| SGLT-2 inhibitor, N (%) | 66 (7.8) | 38 (9) | 28 (6.7) | .101 |
| GLP1 agonist, N (%) | 13 (1.5) | 9 (2.1) | 4 (1) | .176 |
| DOAC, N (%) | 55 (6.5) | 31 (7.3) | 24 (5.8) | .187 |

DOAC= direst oral anticoagulant; GLP1=glucagon like peptide 1; SGLT2= sodium-glucose transporter2
